# Contextualizing wild cereal harvesting at Middle Palaeolithic Ghar-e Boof in the southern Zagros

**DOI:** 10.1038/s41598-024-69056-5

**Published:** 2024-08-13

**Authors:** Simone Riehl, Doğa Karakaya, Mohsen Zeidi, Nicholas J. Conard

**Affiliations:** 1 Senckenberg Centre for Human Evolution and Palaeoenvironment at the University of Tübingen, Hölderlinstrasse 23, 72070 Tübingen, Germany; 2 Institute for Archaeological Sciences, University of Tübingen, Hölderlinstrasse 12, 72070 Tübingen, Germany; 3 Abteilung für Ältere Urgeschichte und Quartärökologie, Institut für Ur-und Frühgeschichte und Archäologie des Mittelalters, Universität Tübingen, Schloss Hohentübingen, 72070 Tübingen, Germany; 4 Department of Cultures, Faculty of Arts, University of Helsinki, Fabianinkatu 24A, 00014 Helsinki, Finland

**Keywords:** Archaeology, Coevolution

## Abstract

A stratigraphic sequence from Ghar-e Boof, a cave site in Iran, covering a period of c. 80,000–30,000 BP and containing more than 20,000 seed and chaff remains, allows a detailed study of the use of annual seed species of Palaeolithic hunter-gatherer groups and its evolution under the influence of changing environmental conditions. Taxonomic changes in the archaeobotanical assemblage and the stable carbon isotope data of pistachio support a considerable change in environmental conditions over the sequence from MIS 5a to MIS 3. The exceptional dominance of wild ancestors of modern crop species, including glume wheat and large-seeded legumes from Middle Palaeolithic layers AH VI (OSL ranges 72–81 ka BP), coincides broadly with the transition from MIS 5a to MIS 4. With the beginning of MIS 4 these taxa are strongly reduced, corresponding with a strong decrease in global CO_2_ concentrations and in the Δ^13^C values of *Pistacia khinjuk*/*atlantica* from the site. Wild glume wheat completely disappears after Middle Palaeolithic AH Vb and never reappears at the site. We hypothesize that the Middle Palaeolithic niche that allowed the harvesting and consumption of wild cereals and legumes ended with a destabilization of the vegetation in early MIS 4.

## Introduction

The concept of a significant contribution of plant-based foods to early hunter-gatherers diets can be considered established on the basis of archaeological, isotopic^1^ and archaeobotanical^2^ evidence. Numerous studies of the role of plants in Palaeolithic hunter-gatherer groups are based on microbotanical evidence from starch and phytolith remains in sediments, dental calculus, and stone tools^3–9^. These studies infer dietary habits, seasonal variations in diet, and the importance of plants in the overall subsistence strategy of anatomically modern and archaic humans, and reveal also that Neanderthal dietary ecology was more complex than previously thought^7^. For some of these sites plant processing tools, such as grinding stones, mortars, and pestles provide supporting evidence for processing practices of plant raw materials. There is, however, still relatively little macrobotanical evidence documenting the collection of wild ancestors of cereals and legumes^10–12^. The discovery of large quantities of wild cereals in Middle Palaeolithic hunter-gatherer contexts at Ghar-e Boof therefore raises questions about the demarcation between the plant-based diet of Middle Palaeolithic humans and the much later Epipalaeolithic hunter-gatherers, who are commonly assumed to be distinct in their harvesting of annual grasses^13,14^.

There is general agreement that the gathering and processing of wild cereals involved a combination of environmental and technological preconditions to guarantee the successful collection of these free-shattering species^15–17^. The short life cycle of grass seeds, which allows a rapid turnover, and the macro-nutritional advantage of seeds, which have a higher caloric density compared to most leaves, roots, and fruits, makes their collection highly attractive to humans, in particular in the face of the decreasing availability of other common food resources^18,19^. In this context, appropriate collection, storage and processing techniques become increasingly important. Only later, with the process of cultivation did desirable traits of seed plants, such as large seed size, high yield potential, and non-shattering seed heads, become targets for domestication^20^, which altogether would have required considerable systematic human intervention, and specific social and cultural practices. Researchers often sought explanations for why these later processes developed primarily in the role of climate, ecology, the cognitive evolution of the human brain, socio-cultural developments and demography^21–42^.

**Figure 1:**
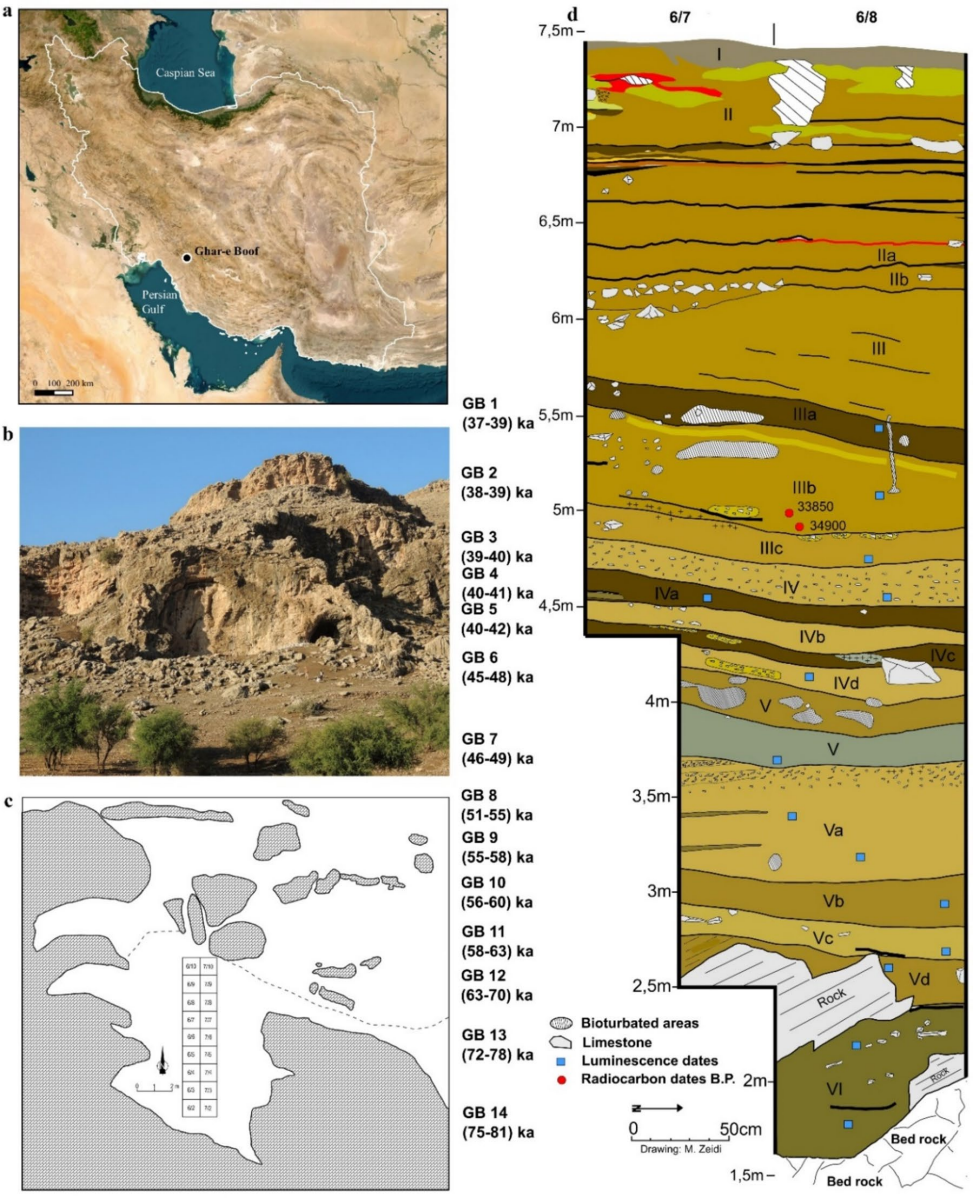
(**a**) Location of Ghar-e Boof (GB) in Iran; map created by M. Zeidi using free open source QGIS software version 3.10.12. https://qgis.org/en/site/, (**b**) Site view; Photo by N. J. Conard, (**c**) Site plan with excavated squares (modified after Ghasidian 2014^46^ and Conard and Ghasidian 2011^49^), (**d**) stratigraphic profile of the archaeological layers AH VI-I and their OSL (GB 14–1) and radiocarbon ages from AH IIIb; by M. Zeidi using free open source Inkscape software version 0.92. https://inkscape.org/.

Ghar-e Boof (N 30.2839°, E 51.4352°) is a cave located in the Dasht-e Rostam Plain, Fars Province, in the southern Zagros Mountains of Iran, which we excavated in 2006, 2007, 2015 and 2017^43–45^. Our team uncovered six main geological and archaeological horizons and 13 sub-horizons representing multiple cultural entities (Fig. 1). The archaeological horizons AH IIIa to IVb yielded calibrated radiocarbon ages of 37–42 ka and are provisionally assigned to the Rostamian of the early Upper Palaeolithic^43–46^. These are underlain by strata intermediate to the Upper and Middle Palaeolithic zones (AH IVc, IVd) yielding OSL dates of 45–48 ka (for chronological overview see also Fig. S1). The underlying Middle Palaeolithic sequence AH V and AH VI yielded ages of 46–81 ka and can be stratigraphically subdivided into AH V with OSL dates between 46 and 49 ka, AH Va-c between 51 and 63 ka, AH Vd between 63 and 70 ka, and AH VI between 72 and 81 ka, placing the entire sequence in the MIS 5a-3. The presence of both archaic and modern human forms is possible at Ghar-e Boof, especially in light of the recovery of skeletal remains of early *Homo sapiens* in the southern Levant^47^ and the temporal overlap of Neanderthals and anatomically modern humans (AMH) in the wider region^48^. However, no human remains have been found at Ghar-e Boof. In line with the documented association with Neanderthal remains in Middle Palaeolithic assemblages in the Zagros, especially at Shanidar, we hypothesize that Neanderthals also made the Middle Palaeolithic assemblages at Ghar-e Boof. While the knappers of the Middle and Upper Palaeolithic used the same local raw materials, the lithic industries of the Middle Palaeolithic horizons are characterized by flake-dominated assemblages and contrast with the rich early Upper Palaeolithic Rostamian assemblages we have provisionally named the Rostamian with diminutive laminar technology. We assume that modern humans made the Upper Palaeolithic assemblage, as has been demonstrated in many regions of western Eurasia.

Detailed palynological studies going back to the Middle Palaeolithic are limited in the wider Zagros region, with the exception of Lake Urmia^50^ about 1000 km NNW of Ghar-e Boof. The pollen sequence from Lake Urmia before 29,000 cal. BP has been indirectly attributed to marine isotopic stages based on the ratio of arboreal to non-arboreal pollen, with the arboreal pollen mainly derived from oak^50^. Since there are few pollen types with long-distance dispersal, such as pine, the Lake Urmia records are of limited validity for assessing the vegetation around Ghar-e Boof. Similarly, the global climate record can only provide a rough approximation for the changing environment at Ghar-e Boof.

Nonetheless, based on these records, the occupation phases of Ghar-e Boof fall within a pronounced sequence of climatic fluctuations with a decrease in high-latitude northern hemisphere insolation at ca. 70–80 ka^51^, which coincides with the transition from the warm interglacial period of MIS 5a to the glacial period of MIS 4, and largely corresponds to AH VI at Ghar-e Boof. In considering ancient environmental conditions, we refer to atmospheric CO_2_ concentrations, which indirectly reflect temperatures and biomass productivity^52,53^. These concentrations can be directly compared to our stable carbon measurements on pistachio remains. Atmospheric CO_2_ concentrations are highly variable throughout MIS 4 and 3, but remain at a consistently low level of 225–190 ppm^54^, during the occupation of AH Vd-III at Ghar-e Boof (Figs. S2 and S4).

The archaeobotanical findings at Ghar-e Boof allow a detailed analysis of the use of wild cereals and legumes by ancient humans as early as 80.000 years ago and contribute to assessing circumstances and processes that lead to their disappearance with the end of the Middle Palaeolithic.

## Results

### Diachronic changes in the composition of the archaeobotanical assemblage of Ghar-e Boof

**Figure 2:**
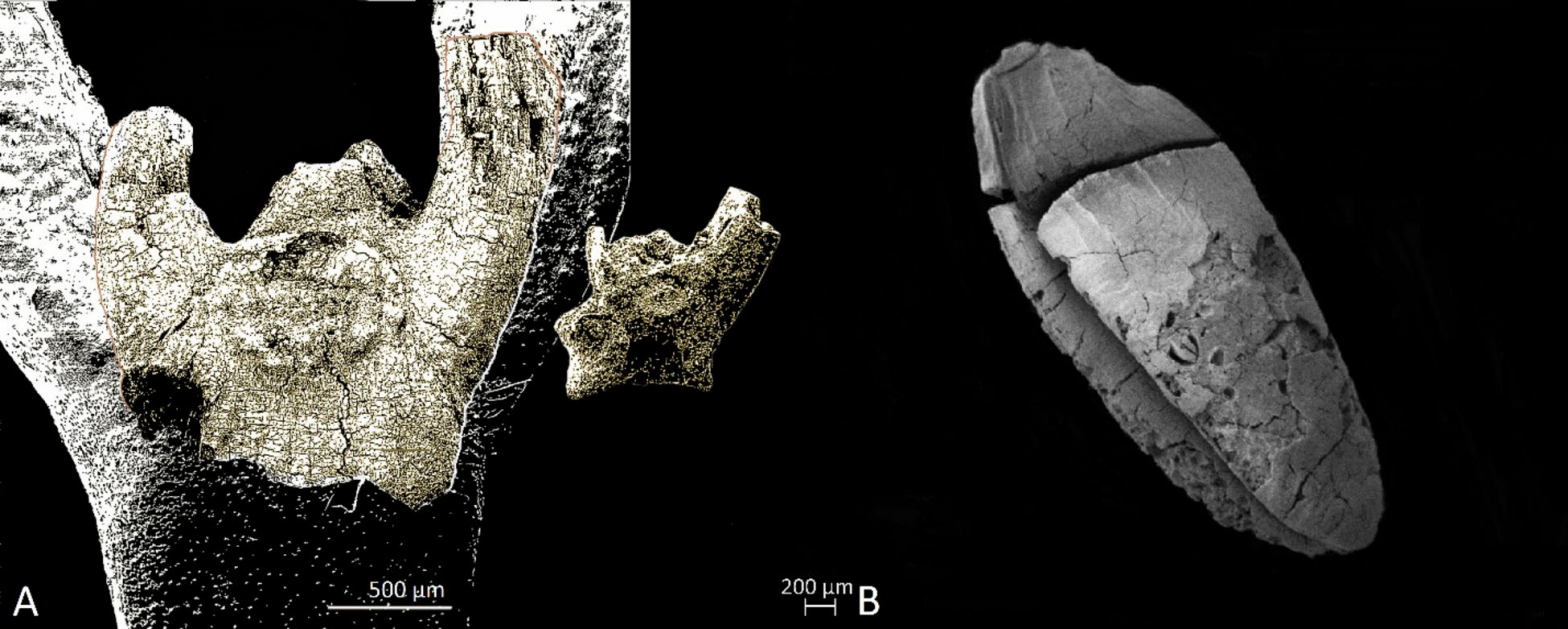
(**A**) Size relations of three different spikelet forks of glume wheat: The largest black and white spikelet fork in the background shows a modern uncarbonized specimen; The sepia-colored specimen in front of the modern spikelet is a 10.000 years old carbonized spikelet fork from aceramic Neolithic Chogha Golan (Central Zagros Mountains); The smallest sepia-colored spikelet fork to the right derives from AH VI at Ghar-e Boof and is about 80.000 years old; (**B**) glume wheat grain from AH VI at Ghar-e Boof. Photo by S. Riehl.

The extraordinary archaeobotanical richness of the Middle Palaeolithic sediments is best expressed by comparing find densities, which are low in the Upper Palaeolithic AH III–IVb with an average of 0,1–2 archaeobotanical records per liter of sediment (see SI data file 1 online). AH V-Vb find densities are similarly low and dominated by pistachio (*Pistacia khinjuk/atlantica*) shell fragments. Glume bases and spikelets of wild glume wheat (*Triticum boeoticum/dicoccoides*) are present in the Middle Palaeolithic AH Vb-Vd with very low numbers. The average density of records in AH VI in contrast is on average 186 per liter of sediment. The taxonomic diversity is also highest in this level. The majority of the finds are glume bases and spikelets of wild glume wheat (*Triticum boeoticum/dicoccoides*), adding up to 18.697 complete spikelets in a total of 10 samples, and represent 95% of all plant remains (Fig. 2). The additional presence of other Poaceae, including their chaff remains supports the assumption that wild grasses were intensively gathered and processed for consumption by the Middle Palaeolithic hunter-gatherers of AH VI.

**Figure 3:**
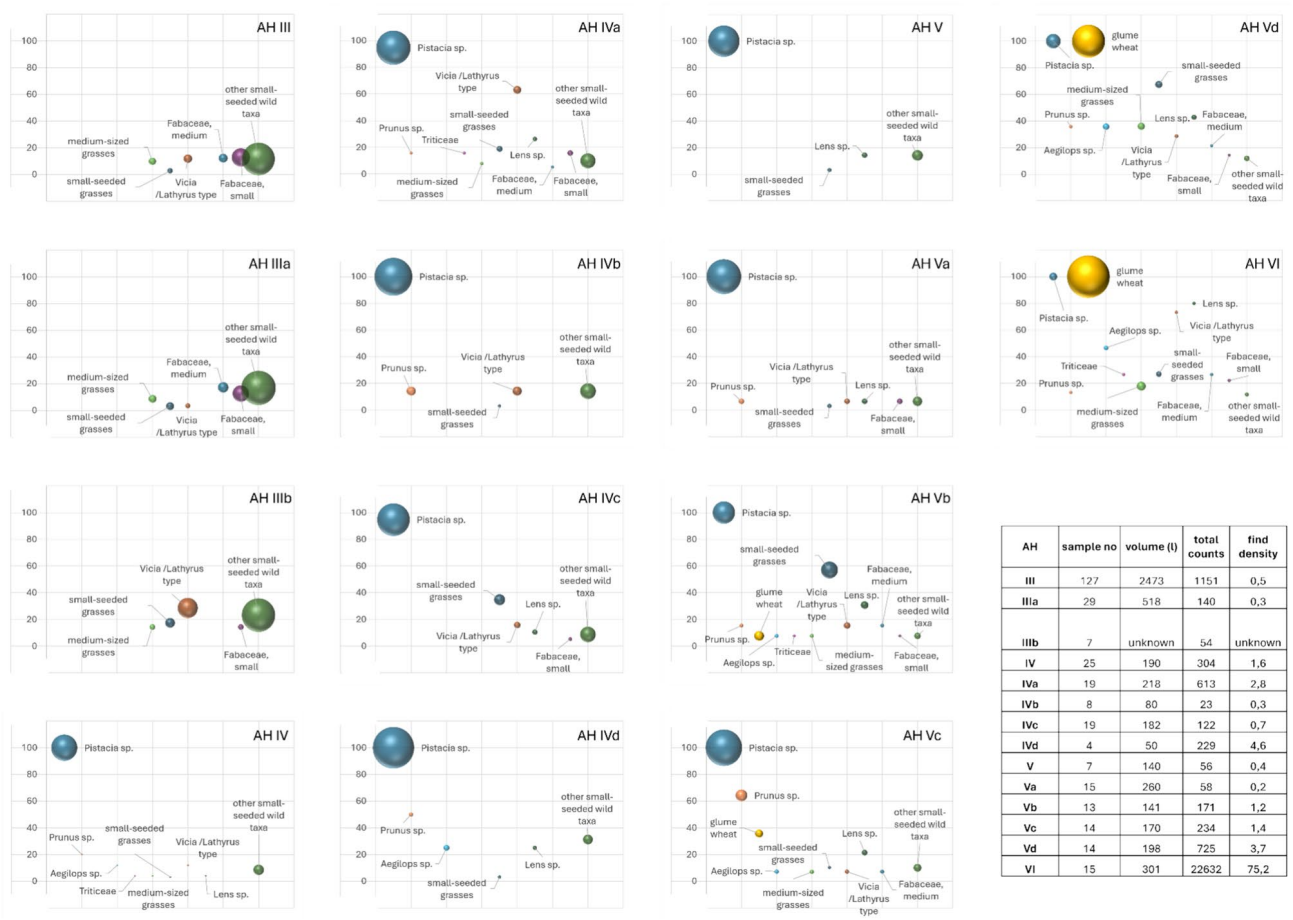
Major taxonomic plant categories (spheres) per archaeological horizon (AH VI-III): The position of a sphere along the y-axis indicates its ubiquity within an archaeological layer, the size of the spheres indicates the relative percentage proportion of the taxonomic category.

Several striking fluctuations in taxonomic proportions and ubiquities are to be noted (Fig. 3). Glume base fragments of wild glume wheat as well as their grains are most numerous in AH VI, slightly decrease in AH Vd and then disappear subsequently from later assemblages, whereas pistachio shell proportions are particularly low in AH VI and Vd, strongly increase from AH Vc onwards and become virtually absent in the earlier layers of the Upper Palaeolithic between 35 and 39 ka (AH III, IIIa and IIIb). Pistachio shell fragments are fluctuating over the complete sequence of archaeological layers AH Vc to AH IV, and if their proportions can be assumed to reflect the availability of the tree or shrub in the landscape, these fluctuations might also be partially related to the changing CO_2_ levels, i.e., a reduction in pistachio stands with decreasing temperatures throughout MIS 4 and 3 (Fig. S2).

While wild legumes, such as lentil (*Lens* sp.) and a large-seeded vetch/grass pea type (*Vicia/Lathyrus* type) occur in AH VI in only small proportions they reach ubiquities of up to 70%. Lentil continues to be present in low proportions and moderate ubiquities until including AH IV, whereas vetch/grass peas species, after lacking in AH V and IVd become very abundant in the Upper Palaeolithic AH IIIb and reach also high ubiquities in AH IVa. Small-seeded grasses (mainly *Eragrostis* sp.) alternate with the wild glume wheat remains in that they increase in proportions and ubiquity with the decrease of wild glume wheat throughout AH VI-Vb, a phenomenon that has also been observed at later sites in the region (e.g., at Chogha Golan^14^). Among other taxa specifically rich in finds in AH VI are cist or rock roses (*Cistus/Helianthemum*) with a major distribution area in Mediterranean landscapes with shrub-steppe habitats.

### Contrasting changes in wild glume wheat dimensions over time

Seed size is among the most frequently discussed phenological traits in seed species as an environmental variable^55,56^, and also as part of the domestication syndrome^57,58^. The recovered Middle Palaeolithic glume wheat grains from Ghar-e Boof AH VI are comparable in size to aceramic Neolithic grains from other Near Eastern sites, including those from Chogha Golan^59,60^ (width increase is only 4% compared to Ghar-e Boof, whereas spikelet width is increased for 112%; see Fig. S3). A conspicuous lack of congruency in grain sizes and spikelet dimensions of the glume wheats at Ghar-e Boof can be related to differential charring reactions of grains and chaff in dependence of charring temperature. Referring to the grain dimensions, the spikelets from Ghar-e Boof are comparatively small (Fig. 2). Experimental charring revealed that grains of the glume wheats undergo a general length shrinkage, but an up to 38% width increase^61^ at temperatures between 250 and 300 °C^62^, whereas wild emmer indicates a decrease of spikelet width ranging between 12 and 20% (^60^ supplementary file therein). Experimental charring indicates that starch, the major component of grains, accounts for grain size alterations during carbonization^63^. Given that the starch component of grains is at least partially responsible for grain expansion in breadth, known as the biochemical process of gelatinization, the shrinkage of spikelets, being composed of mainly cellulose, becomes comprehensible, and at least partially explains virtual size discrepancies of grains and spikelets.

However, the size difference of the Middle Palaeolithic and Neolithic spikelets represents a real difference in the size of the plants, requiring further consideration in the light of plant evolution and atmospheric CO_2_ concentrations. Because of the absence of cultivation and domestication, we hypothesize that the size increase is related to differences in atmospheric CO_2_ concentrations and natural adaptation processes of the plants (Fig. 2).

### Stable carbon isotope ratios and environmental conditions

**Figure 4:**
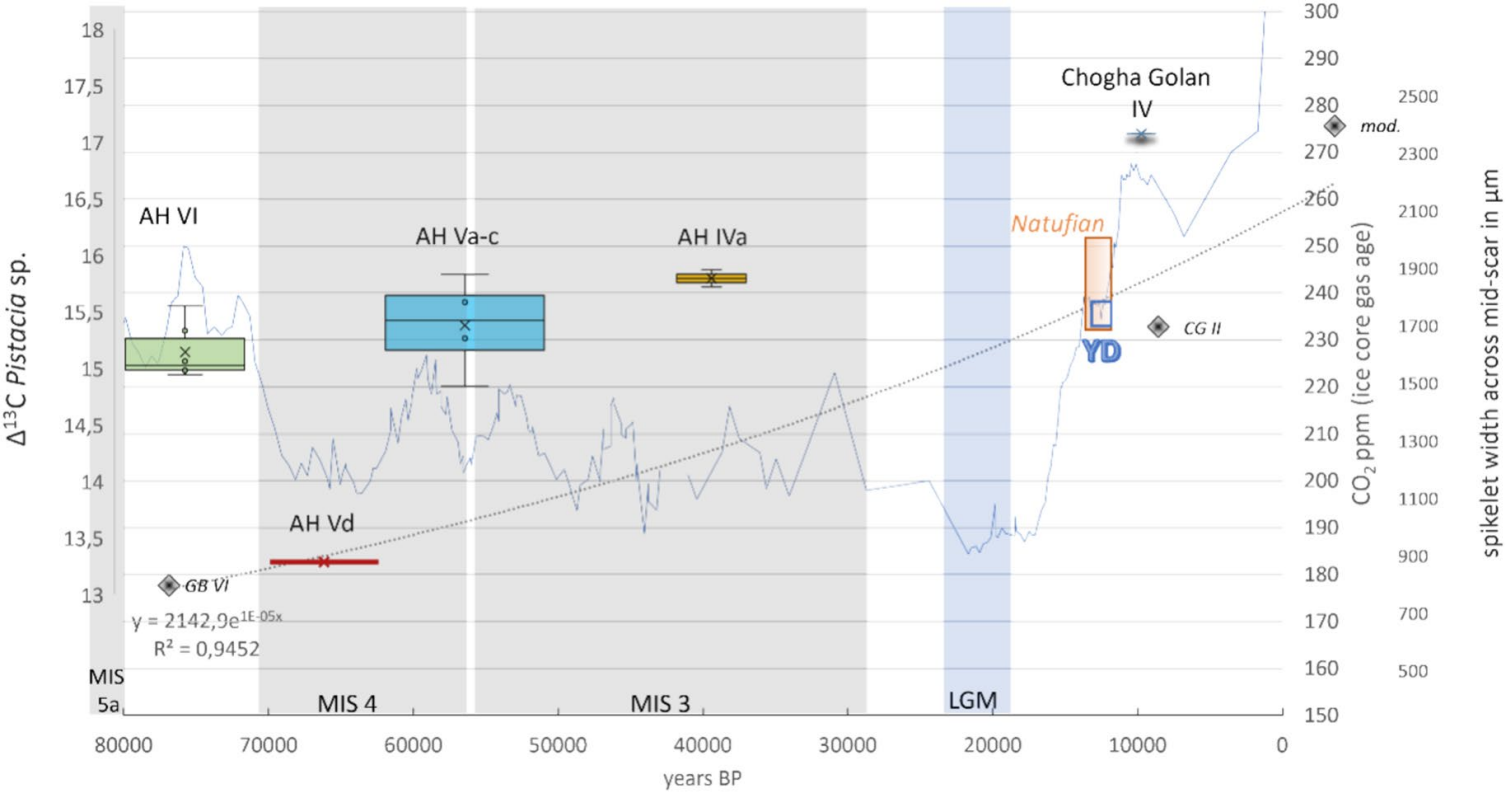
CO_2_ fluctuations over the last 80.000 years (raw data downloaded from NOAA.gov, data sources: 80.000–34.000 BP^54^, 34.000–21.600 BP and 9.000–5.000 BP^64^, 21.600 BP-9.000 BP^65^) and Δ^13^C values of *Pistacia khinjuk/atlantica* in different archaeological horizons (AH) at Ghar-e Boof and Chogha Golan IV; Spikelet widths of glume wheats at both sites are also shown (diamonds).

The Δ^13^C values of *Pistacia khinjuk/atlantica* from Ghar-e Boof correlate well with global CO_2_ fluctuations throughout the archaeological sequence (Fig. 4; for details on the methods, see Methods section). Particularly striking is the sharp decrease in global CO_2_ levels with the end of MIS 5a, followed by very low Δ^13^C values of pistachio with the beginning of MIS 4, which coincides with AH Vd at Ghar-e Boof. Subsequent occupation horizons, which fall within a sequence of strongly fluctuating CO_2_ levels, produced higher Δ^13^C values again, and thus, considering the chronological imprecision of such wide time frames dated with OSL, probably reflect occupation events coinciding with periods of elevated CO_2_ levels. The Δ^13^C values of pistachios from the Chogha Golan tell site (AH IV) also reflect well the elevated CO_2_ concentrations during the aceramic Neolithic era. Apart from the general correlation of Δ^13^C values of *Pistacia khinjuk/atlantica* with CO_2_ concentrations, the stable carbon isotopes also suggest a comparatively high level of stress for pistachio trees during the Ghar-e Boof AH Vd sequence, which may have been related to reduced moisture availability.

## Discussion

In order to assess the environmental context of Middle Palaeolithic hunter-gatherers harvesting wild wheat, it is essential to consider the changing concentrations of atmospheric CO_2_, climate, and vegetation throughout the complete occupation history of Ghar-e Boof.

Plant physiologists and palaeoclimatologists have been studying the close relationship between CO_2_ fluctuations, vegetation growth and temperatures since at least the 1970s, and more recently in the context of global warming. Leaving aside other gases contributing to the greenhouse effect and positive feedback mechanisms, and focusing on changes in orbital parameters, variations in solar radiation, volcanic and tectonic activity during the Pleistocene, the simplified interpretation of low CO_2_ concentrations and cooler conditions can help to consider climate dynamics in a region with few local palaeoclimate proxies. We use these data to gain a general insight into fluctuations in temperature, biomass production, and to provide a baseline for comparison with our Δ^13^C measurements of pistachio shells during the occupation of Ghar-e Boof. Similarly, pollen cores from lakes Urmia and Zeribar, about 1000 km NNW of Ghar-e Boof, can provide a general overview of vegetation trends in the wider geographical region of Iran.

The pollen sequence from Lake Urmia before 29,000 cal. BP has been indirectly correlated to marine isotopic stages based on the ratios of arboreal and non-arboreal pollen, with the arboreal pollen mainly derived from oak^50^. MIS 5a, a still warm period attributed within this sequence to the interstadial Kaboudan II, transitions sharply into the MIS 4 glaciation (71–57 ka) over a comparatively short period of only 12,000 years, which has been associated by some scholars with a decline in high-latitude northern hemisphere insolation at ca. 80–70 cal ka BP^51^. Based on the OSL dates from Ghar-e Boof, this shift coincides with the occupation period AH VI at Ghar-e Boof between ca. 81–72 ka OSL. CO_2_ concentrations at this time are still relatively high with values between 225 and 250 ppm, which were only reached again about 70,000 years later at the end of the Pleistocene, a phase that can be climatically characterized as Mediterranean in the region, with mild, wet winters and hot, dry summers. The taxonomic composition of the seed assemblage in AH VI at Ghar-e Boof support a Mediterranean climate. In addition to the presence of many wild ancestors of modern crops (*Triticum boeoticum/dicoccoides*, *Lens* sp., *Lathyrus/Vicia* and *Aegilops* sp.), other typically Mediterranean taxa such as *Cistus/Helianthemum* and several Lamiaceae species are present in the archaeobotanical assemblage. In the pollen sequence from Lake Urmia, the transition between MIS 5a and MIS 4 is characterized by the almost complete disappearance of oak, which was still present with about 20% in MIS 5a. The percentage of oak pollen continues to fluctuate with low values (< 10%) during MIS 4 and 3 and does not reach higher levels again until the middle Holocene. In contrast, non-arboreal pollen, mainly represented by Poaceae, increases strongly with the end of MIS 5a (from 30 to > 70%), making grass populations a dominant vegetation type at the time of the occupation of Ghar-e Boof VI, and this could help to explain the high abundance of collected wild *Triticum* species. With the beginning of MIS 4 atmospheric CO_2_ concentrations decrease strongly, which is also reflected in the Δ^13^C values of pistachio in AH Vd (ca. 63–70 ka; Fig. 4), and probably reflects the cooling that characterizes the transition from MIS5a to MIS 4, which led to the decline of oak pollen at Lake Urmia. The pollen of Urmia Poaceae also decreases to 55–25% in contrast to Artemisia pollen, which increases to about 40%, indicating increasingly arid and probably also cooler conditions during the Ghar-e Boof AH Vd compared to the previous AH VI.

Climatic instability during MIS 4 was considerable, with changes in temperature and precipitation patterns likely occurring on relatively short timescales, resulting in shifts in ecosystems and habitats. This may be reflected in the extensive taxonomic change in the archaeobotanical assemblage by the end of AH Vd. Wild glume wheat disappears and the ubiquity of large-seeded legumes decreases sharply. This likely reflects a striking change in vegetation that had a direct impact on the foraging and dietary spectrum of the Middle Palaeolithic hunter-gatherers of Ghar-e Boof. The faunal assemblage is difficult to interpret in this way due to the small number of animal bones in Ghar-e Boof AH Vd. A striking pattern, however, is that bones of medium-sized ungulates and sheep and goats reach their highest numbers in AH VI and then virtually disappear in AH Vd (^66^, Suppl. Inf.).

Most of the following occupational levels (AH Va, V, IVd-a, IV, IIIc-a, III) covering a sequence from the final Middle Palaeolithic into the Upper Palaeolithic fall into MIS 3, an intermediate period between the more extensive glaciation of MIS 4 and the peak of the Last Glacial Maximum (LGM) in MIS 2. Shifts in vegetation patterns and animal distributions in response to changing climatic conditions continued to be similar in magnitude to the previous MIS 4. The taxonomic diversity of the archaeobotanical assemblage at Ghar-e Boof remains low throughout these occupation phases, with a major component of pistachio between AH Va and AH IV, and an increased ubiquity and proportion of large-seeded legumes in AH IV and subsequent phases. A drastic reduction of rodents in the small vertebrate assemblages, roughly representing the layers of AH IV, shows a more general pattern of a short period of colder and/or drier conditions during the shift from the Middle Palaeolithic to the Upper Palaeolithic^67^. The uppermost archaeological horizons of II and I, although containing Epipalaeolithic artifacts, have been partially contaminated with Islamic period plant remains through the actions of burrowing animals and are not considered in this study.

The most important food plants of the Middle and Upper Palaeolithic hunter-gatherers at Ghar-e Boof, in terms of proportion and ubiquity, were large-seeded Fabaceae, pistachio, and Poaceae, especially wild glume wheat during the Middle Palaeolithic occupation of AH VI.

Although the nut kernels are edible after parching and roasting, the wild pistachio (*Pistacia atlantica*), known as *Baneh*, is now economically important in many rural areas of Iran, mainly for its resins, named *Saqez*^68^. Wild pistachio stands can occupy a wide range of habitats, including cold to moderate semi-arid climates, from woodland to desert, due to their resistance to adverse environmental conditions. In the Zagros, they grow at altitudes between 1000 and 2300 m a.s.l. A recent study showed that the effective climatic limit for wild pistachio is 180 mm of mean annual precipitation, with mean minimum temperatures not lower than 6 °C and mean maximum temperatures not higher than 33 °C. The main growth-limiting factor, however, is shallow soil depth, which reduces the storage capacity of soil moisture, that is necessary for the survival of wild pistachio during drought and prolonged dry periods^69,70^. Since we can assume that the climatic conditions during the transition sequence from MIS 5a to MIS 4 were sufficiently favorable for pistachio growth, two options for explaining the low pistachio proportions in AH VI and Vd are that pistachio was generally not widespread in the region, or it was not of high interest as a food source for ancient humans. Environment-based probability distributions of different *Pistacia* species highlight their long-term evolutionary history and confirm their sensitivity to precipitation in the driest month and isothermality, i.e., the evenness with which temperatures are distributed throughout the year compared to variations within a single day^71^. In the Urmia pollen diagram, pistachio appears with higher percentages until MIS 5c and then practically disappears from the pollen diagram. Nevertheless, the plant apparently produced a large number of fruits about 1000 km to the south, which were collected in varying quantities by Ghar-e Boof gatherers during the occupations of AH Vc through AH IV. It is unclear whether the pistachio shrubs or trees disappeared completely in AH III or whether this absence represents a recording bias. Today, pistachio trees are absent in the immediate region and the main woody vegetation component is oak^72^.

In addition to the uniqueness of thousands of wild wheat glume base fragments in Middle Palaeolithic deposits, their small dimensions raise questions about the evolution of wild glume wheat in this geographic region. As previously stated, the discrepancy in the increase of dimensions observed between chaff and grain is primarily attributed to taphonomic factors, namely differences in the rates of shrinking and expansion of both object types upon charring.

Seed size increase in later periods at the end of the Pleistocene is usually attributed to the domestication syndrome^57^, and as such is explained by a continuous plant-human coevolution that probably began even before the Epipalaeolithic. The lack of measurable Pleistocene archaeobotanical records has so far hindered the study of wild cereal seed size evolution beyond the onset of emerging cultivation.

Despite the measurable presence of 20–60% grain size increases during the 2000–4000 year long domestication episode of cereals^57,73^, the seed size increase of glume wheat comparing the Middle Palaeolithic Ghar-e Boof and the aceramic Neolithic Chogha Golan is only 4% (Figs. S3 and 2). It is safe to assume that no continuous human influence acted on the wild cereals growing in the region. However, the small differences between the Middle Palaeolithic and the probably already manipulated aceramic Neolithic grains, suggest that the evolution of plant size is a complex matter with multiple variables^55^. These include seed size/seed number trade-offs that define plant fitness and phenotypic plasticity in relation to climate change^56^. Consequently, some researchers call for more comprehensive approaches that can test the importance of different potential drivers simultaneously^58,74^.

Notwithstanding its evolutionary multivariability, researchers have proposed explanations for the increase in seed size. Those working in the context of neolithization sometimes suggest that larger seeds have advantages in certain types of competition, such as deeper burial in soils disturbed by human tillage^75^, not at least based on the fact that modern domesticated cereals show higher germination rates, seedling and total plant size, e.g., 40% larger final plant size^58,76^. However, recent experimental studies suggest that deeper seed burial under cultivation is unlikely to be a general mechanism of selection for increasing seed size^77^. Other researchers with a long-term ecological focus argue that species whose seedlings can establish themselves in the shade of forest canopies tend to have larger seeds^55^. From the perspective of the vegetation cover this would represent an opposite ecological habitat type compared to disturbed soils, which are mostly found in open landscapes.

In their seminal paper on the effects of low atmospheric CO_2_ on plants, Sage and Coleman describe how CO_2_ acted as a significant evolutionary agent and affected the primary productivity of plants^78^. Even at optimal temperatures for C3 photosynthesis, i.e. 20–30 °C, the reduction of atmospheric CO_2_ from 360 to 180 ppm results in reduced photosynthetic capacity and about 50% reduced biomass production, a fact that Sage considered causal for the limitations in the beginnings of agriculture^79^. With the end of the AH VI sequence at Ghar-e Boof, global CO_2_ concentrations dropped from 240 to 180 ppm, with a mean of 200 ppm, reaching levels similar to those of AH VI only at the beginning of the Younger Dryas^80^ (Fig. 4). Experimental wheat growth under reduced CO_2_ concentrations shows relative responses in seed yield of 0.6 at 240 ppm and less than 0.4 at 200 ppm^81^. The documented global changes in atmospheric CO_2_ over the past 80,000 years likely caused dramatic fluctuations in wild wheat populations and other C3 dominated plant communities and could explain the absence of wild cereal species in the later Middle Palaeolithic and Upper Palaeolithic sequence at Ghar-e Boof.

Although the lack of skeletal remains at Ghar-e Boof precludes a definitive assignment of the archaeological layers to specific human forms, we hypothesize on the basis of artefacts, OSL, and radiocarbon dating that Neanderthals made the Middle Palaeolithic assemblages in the oldest layers (AH VI) and modern humans made the early Upper Palaeolithic assemblages (AH IIIa to IVb). For the intermediate horizons AH IVc and IVd, no statement is made at this time. Since our extensive archaeobotanical data set allows us to consider the temporal development of the plant diet, the differences between the oldest Middle Palaeolithic layers (AH VI), the later Middle Palaeolithic and the Upper Palaeolithic layers (AH IIIb-III) are particularly significant. Our data show a development of the use of predominantly wild glume wheat alongside a variety of legumes in the oldest Middle Palaeolithic, through high proportions of pistachios in the transitional layers to large quantities of legumes in the upper layers of the Upper Palaeolithic. While no ground stone tools have been found to support a systematic and regular exploitation of the glume wheats by the earliest occupants of the site, their presence in thousands and the ease of releasing grain from the spikelets makes their role as an important dietary component highly likely. Since wild plants shed their seeds upon maturity they must have been collected before they were fully ripe. Simple drying, either in the sun or near a fire, would have released the grains for either direct consumption or further preparation into processed foods. Since the spikelets were charred, it is likely that they were discarded after the seeds were released. These archaeobotanical findings from Ghar-e Boof AH VI place the evidence for the plant component of the diet in line with the Neanderthal sites of Shanidar Cave^10,82^ in Iraq and Kebara^11^ and Amud Caves^4^ in Israel. Starch grains and phytoliths of the Poaceae were found in Shanidar and Amud, while at Kebara a broad spectrum of charred seed remains, dominated by legumes and pistachio were identified. At Shanidar, the technique of cooking was based on damage to the starch grains^82^ and on charred food remains, which were interpreted as ground legumes^10^.

The researchers of the archaeobotanical remains at Amud and Kebara have reached the conclusion that “the records concur that exploitation of a broad spectrum of food resources was part of the Palaeolithic lifeways long before it became the foundation of, and a prerequisite for, an economic revolution”^4^. This indicates “that it was not revolutionary insights into the economic potential of Levantine vegetal resources which led to resource intensification in the region c. 12 kyr ago”^4^. This observation, which is more than 20 years old, is repeated in many of the subsequent archaeobotanical investigations of Middle and Late Palaeolithic sites and is now evident with particular clarity in the findings at Ghar-e Boof, where, in addition to the wide range of plant remains, wild forms of later cultivated plants are also found in large numbers.

A comparison of a substantial number of phytolith and starch records from various Neanderthal and *Homo sapiens* contexts revealed a pronounced general contribution of the Triticeae to the diets of these hominins. The authors also conclude that the dietary ecology of Neanderthals was more complex than previously assumed^7^. The authors argue against the widely held view that the main reason for Neanderthal extinction was that their diet was more limited than that of modern humans, and that it was unbalanced in terms of calories and nutrients^7^. On the other hand, factors such as new technologies and the adaptations that associated with the development of symbolism also played a role in the demographic expansion of modern humans.

Nevertheless, plant foods probably contributed to population dynamics at the transition from the Middle to the Upper Palaeolithic in the Zagros. This is because cooler periods and lower CO_2_ levels resulted in lower plant biomass production, making it more difficult to switch to plant resources when game declined. The disappearance of wild wheat from Ghar-e Boof with the drop in atmospheric CO_2_ at the beginning of MIS 4 is consistent with a decrease in plant food sources for Middle Palaeolithic hunter-gatherers. Starting from a high taxonomic diversity at the end of MIS 5a (AH VI), a continuous decrease in taxonomic diversity can be observed in the course of the occupation periods (see also SI data file). We can therefore assume that, related to lowering CO_2_ levels photosynthetic rates in plants dropped and primary productivity was decreased after AH VI leading to the disappearance of wild glume wheats and other taxa adapted to a Mediterranean climate regime. Closely related to this, the decreasing CO_2_ levels might have caused shifts in vegetation types. These changes may have resulted in less available food for herbivores and also affected the types of plants and animals available to Neanderthals, potentially impacting their diet and foraging strategies.

## Conclusions

Consideration of archaeobotanical assemblages throughout the occupation history of Ghar-e Boof, along with changing atmospheric CO_2_ concentrations, has proven informative in assessing the environmental context of Middle Palaeolithic hunter-gatherers harvesting wild wheat. Linking the taxonomic composition and diversity of macrobotanical remains with stable carbon isotope measurements over a long-term sequence reveals changes likely related to climatic fluctuations. Comparison of size differences between 80,000- and 11,000-years-old wild glume wheat suggests that the observed increase in size over this time period may be related to increasing atmospheric CO_2_ concentrations and natural plant adaptation processes.

Fluctuating plant biomass productivity throughout the MIS 5a, MIS 4, and MIS 3 sequence, with decreasing temperatures and atmospheric CO_2_ concentrations, was accompanied by shifts in ecosystems and habitats, reflected in the extensive taxonomic change in the archaeobotanical assemblage. This resulted in very different available plant foods for Neanderthals and *Homo sapiens*. While Middle Palaeolithic hunter-gatherers harvested expanded populations of wild cereals, legumes, and other wild species, the onset of MIS 4 and its significant changes in temperature and atmospheric CO_2_ concentrations reduced common plant food sources. Wild cereals were no longer available in abundance and were increasingly replaced over time by small-seeded grasses and later by pistachio and a few other wild species by Upper Palaeolithic groups, who also supplemented their plant diets with larger-seeded legumes.

The plant consumption patterns of Ghar-e Boof at AH VI have similarities with other Neanderthal sites in Israel and Iran. Neanderthals and *Homo sapiens* had a pronounced general contribution of Triticeae species to their diets, and we argue that the exploitation of a wide range of food resources was a general part of Palaeolithic lifeways^83^.

The study of archaeobotanical remains from Palaeolithic sites and the comparative contextualization of such findings within the framework of the plant-food diets of Neanderthals and *Homo sapiens* contributes to our understanding of the differences and similarities in subsistence behavior of both species and, in the case of Ghar-e Boof, allows insights into the ecological evolutionary history of wild ancestors of modern cereal species under fluctuating climatic conditions.

## Methods

### The archaeobotanical data set

Excavators at Ghar-e Boof floated all of the sediments from the Palaeolithic strata by hand to maximize the recovery of archaeobotanical specimens^83^. A total of 343 archaeobotanical samples (5502 L of sediment) from Ghar-e Boof were analyzed for this study and resulted in 27,465 identified plant remains (SI data file).

Seed and chaff identification was carried out according to morphological criteria using the Tübingen reference collection and a Leica GZ6 binocular. REM images were taken with a LEO model 1450 VP at the Institute of Micropaleontology, University of Tübingen. The taxonomy of the identified plant remains follows archaeobotanical conventions.

Pistachio nutshells were not identifiable to the species level, but because *Pistacia atlantica* and *Pistacia khinjuk* are the dominant wild species in Iran today^70^, we classify the archaeobotanical taxa *Pistacia khinjuk/atlantica*. They were mainly preserved in fragments, therefore they were weighed and converted into whole seeds, with one seed weighing 25 mg. Two halves of the wheat glume bases were added to one spikelet. The raw data set contains 94 archaeobotanical taxa, and the analysis in the last years aimed at an equal representation of samples from all excavated horizons (SI data file).

The uppermost sediment layers of the site, AH II and III, were found to be occasionally contaminated with modern seeds, mainly carbonized crop seeds of modern or late historic times as demonstrated by radiocarbon dating of some of these seeds^84^. Burrowing animals and the biogalleries they create are common in Iran and a degree of contamination from burrowing animals is more the rule than exception in Iranian caves and rock shelters. We critically reviewed the samples from these archaeological horizons for certain and possible contamination and excluded them from the current analysis (marked by ‘C’ and ‘P’ in the SI data file). Most of the AH III samples were not systematically checked for pistachio fragments, although these were much rarer in AH III than in the previous occupation phases. Therefore, AH III was excluded from some of the additional data analyses.

To rule out contamination of the archaeobotanical assemblage from AH VI, and in particular to verify the Middle Palaeolithic age of the glume wheat, we submitted some of the plant remains for AMS radiocarbon dating, expecting them to reach the radiocarbon limits if they were from intact contexts that had previously yielded OSL ranges of 72–81 ka. Since the radiocarbon dates fell at the limit of radiocarbon dating with ages up to 47 ka BP, we conclude that the plants are contemporaneous with the formation of AH VI.

### Quantification of the archaeobotanical assemblage

In order to compare changes in seed and chaff composition across the archaeological sequence, we grouped taxa into broader taxonomic categories that allow for their functional evaluation. For example, *Agrostis* type, *Eragrostis* sp., *Phalaris sp., Poa* sp., and Poaceae, indet. (small-seeded) have been combined into the taxonomic category of small-seeded grasses.

Proportions and ubiquities were calculated for all samples from a given archaeological horizon. While proportions indicate the layer-wise percentage composition of taxonomic categories, thus explaining the abundance of a taxon based on its number of records, ubiquities account for the overall presence of certain taxonomic units within a layer, or in other words, how regularly a taxon occurs in all samples of a single archaeological horizon, regardless of its absolute number of records. These are archaeobotanical standard methods helping to assess the full extent of taxa presence in an archaeological site^85,86^.

### Stable isotopes data

Because of their ability to photosynthesize, green plants can convert sunlight and CO_2_ into chemical energy in their vegetative and reproductive tissues, which, if preserved in geological and archaeological sediments, can be used to extract information about atmospheric CO_2_ concentrations and water availability during the plant’s lifetime. Annual seeds are particularly well suited, as they provide information on these parameters for the relatively short period of seed and fruit formation^87^.

The principles of the relationship between carbon isotope composition and water status of the plant are well established in plant physiology and agronomy^88,89^, and during the last three decades numerous studies have been carried out on archaeobotanical crop species, focusing on ancient agricultural systems (see reviews^87,90,91^). Because δ^13^C_plant_ is directly related to atmospheric δ^13^C (δ^13^C_air_), δ^13^C_plant_ reflects atmospheric CO_2_ concentrations within the extended time frames of climatic oscillations. When considering shorter periods, such as the Holocene, δ^13^C_plant_ in seeds essentially provides a signal for the availability of moisture during the grain-filling period of the plant.

Because pistachio shell fragments are the most ubiquitous and abundant taxon providing the required mass for stable isotope analysis at Ghar-e Boof, we measured pistachio shells in samples from AH VI-IV in the Institute of Geosciences (University of Tübingen). Analysis was conducted using a FinniganMAT252 gas source mass spectrometer with a ThermoFinnigan GasBench II/CTC Combi-Pal autosampler. Prior to mass-spectrometric measurements, the pistachio fragments were treated with 5% HCl to eliminate sedimentary carbonate. The stable isotopes were measured as the ratio of the heavier isotope to the lighter isotope (^13^C/^12^C) and reported as δ values in parts per 1000 or ‘‘per mil’’ (‰) relative to internationally defined standards for carbon (Vienna Pee Dee Belemnite, VPDB). Changes in δ^13^C_air_ during Earth’s history were taken into account by applying a formula that captures the discrimination of the heavy isotope, before comparing δ^13^C_plant_ values in archaeological remains originating from different chronological units, which transforms δ^13^C_plant_ into Δ^13^C values (for details see^87^). There are as yet no comparative data sets for natural Δ^13^C ranges of pistachio because the method is primarily carried out in Holocene agricultural settlements on cultivated plants^92^ and there are still very few studies on perennial plants and woody plants^91^. In general, however, a relative diachronic comparison can be made on the basis of previous research and the development of Δ^13^C in the prehistoric course can be considered. High values indicate little to no drought stress and lower values indicate moderate to pronounced drought stress.

## Supplementary Information

Supplementary Information.

Supplementary Figures.

## Data Availability

The archaeobotanical samples are currently archived at the University of Tübingen. The raw data are available as a supplementary Excel file linked to this work.
