# Author Correction: Contextualizing wild cereal harvesting at Middle Palaeolithic Ghar-e Boof in the southern Zagros

**DOI:** 10.1038/s41598-024-74033-z

**Published:** 2024-10-11

**Authors:** Simone Riehl, Doğa Karakaya, Mohsen Zeidi, Nicholas J. Conard

**Affiliations:** 1 Senckenberg Centre for Human Evolution and Palaeoenvironment at the University of Tübingen, Hölderlinstrasse 23, 72070 Tübingen, Germany; 2 Institute for Archaeological Sciences, University of Tübingen, Hölderlinstrasse 12, 72070 Tübingen, Germany; 3 Abteilung für Ältere Urgeschichte und Quartärökologie, Institut für Ur‑und Frühgeschichte und Archäologie des Mittelalters, Universität Tübingen, Schloss Hohentübingen, 72070 Tübingen, Germany; 4 Department of Cultures, Faculty of Arts, University of Helsinki, Fabianinkatu 24A, 00014 Helsinki, Finland

Correction to: *Scientific Reports*10.1038/s41598-024-69056-5, published online 13 August 2024

The original version of this Article contained an error in the legend of Figure 1.

“(**a**) Location of Ghar-e Boof (GB) in Iran; map created by M. Zeidi using free open source QGIS software version 3.10.12. https://qgis.org/en/site/, (**b**) Site view; Photo by N. J. Conard, (**c**) Site plan with excavated squares, (**d**) stratigraphic profile of the archaeological layers AH VI-I and their OSL (GB 14–1) and radiocarbon ages from AH IIIb; by M. Zeidi using free open source Inkscape software version 0.92. https://inkscape.org/.”

now reads:

“(**a**) Location of Ghar-e Boof (GB) in Iran; map created by M. Zeidi using free open source QGIS software version 3.10.12. https://qgis.org/en/site/, (**b**) Site view; Photo by N. J. Conard, (**c**) Site plan with excavated squares (modified after Ghasidian 2014^46^ and Conard and Ghasidian 2011^49^), (**d**) stratigraphic profile of the archaeological layers AH VI-I and their OSL (GB 14–1) and radiocarbon ages from AH IIIb; by M. Zeidi using free open source Inkscape software version 0.92. https://inkscape.org/.”

The original Article has been corrected.

