# Supplementary material for: Contextualizing wild cereal harvesting at Middle Palaeolithic Ghar-e Boof in the southern Zagros
Source: Sci Rep. 2024 Aug 13;14:18748. doi: 10.1038/s41598-024-69056-5 (PMC11322544; doi:10.1038/s41598-024-69056-5)
Supplement: Supplementary file 2 — Supplementary Figures. [file 41598_2024_69056_MOESM2_ESM.pdf]

# Contextualizing wild cereal harvesting at Middle Palaeolithic Ghar-e Boof in the Southern Zagros

Simone Riehl[1,2]*, Doğa Karakaya[2,4], Mohsen Zeidi[1,3], Nicholas J. Conard[1,2,3]

[1] *Senckenberg Centre for Human Evolution and Palaeoenvironment at the University of Tübingen, Hölderlinstrasse 23, D-72070 Tübingen, Germany*
[2] *Institute for Archaeological Sciences, University of Tübingen, Hölderlinstrasse 12, D-72070 Tübingen, Germany*
[3] *Abteilung für Ältere Urgeschichte und Quartärökologie, Institut für Ur-und Frühgeschichte und Archäologie des Mittelalters, Universität Tübingen, Schloss Hohentübingen, D-72070 Tübingen, Germany*
[4] *Department of Cultures, Faculty of Arts, University of Helsinki, Fabianinkatu 24A, 00014 Helsinki, Finland*

*Correspondence email address: simone.riehl@senckenberg.de

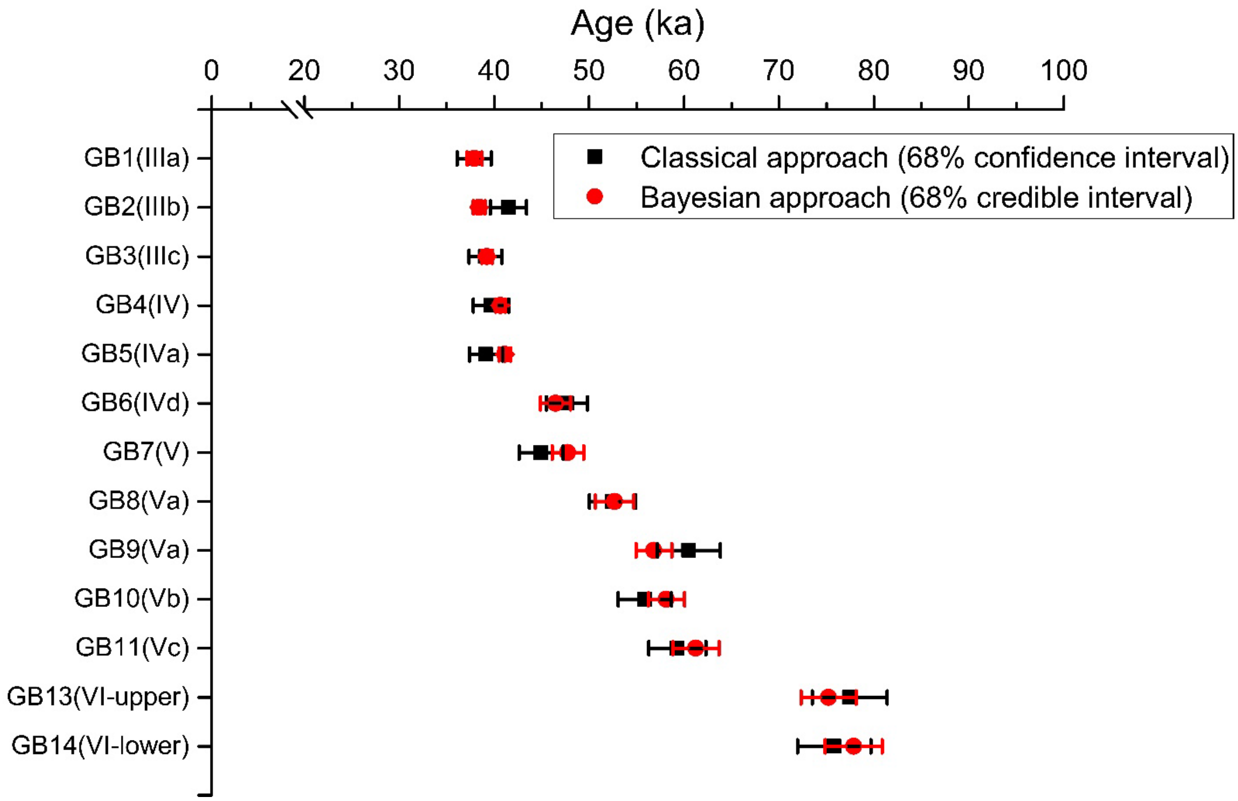


Fig. S1 Optically stimulated luminescence (OSL) chronology of the different archaeological horizons (VI-lower – IIIa) at Ghar-e Boof; Comparison of the quartz OSL ages derived from Bayesian modeling and the classical approach. With permission of Maryam Heydari[1].

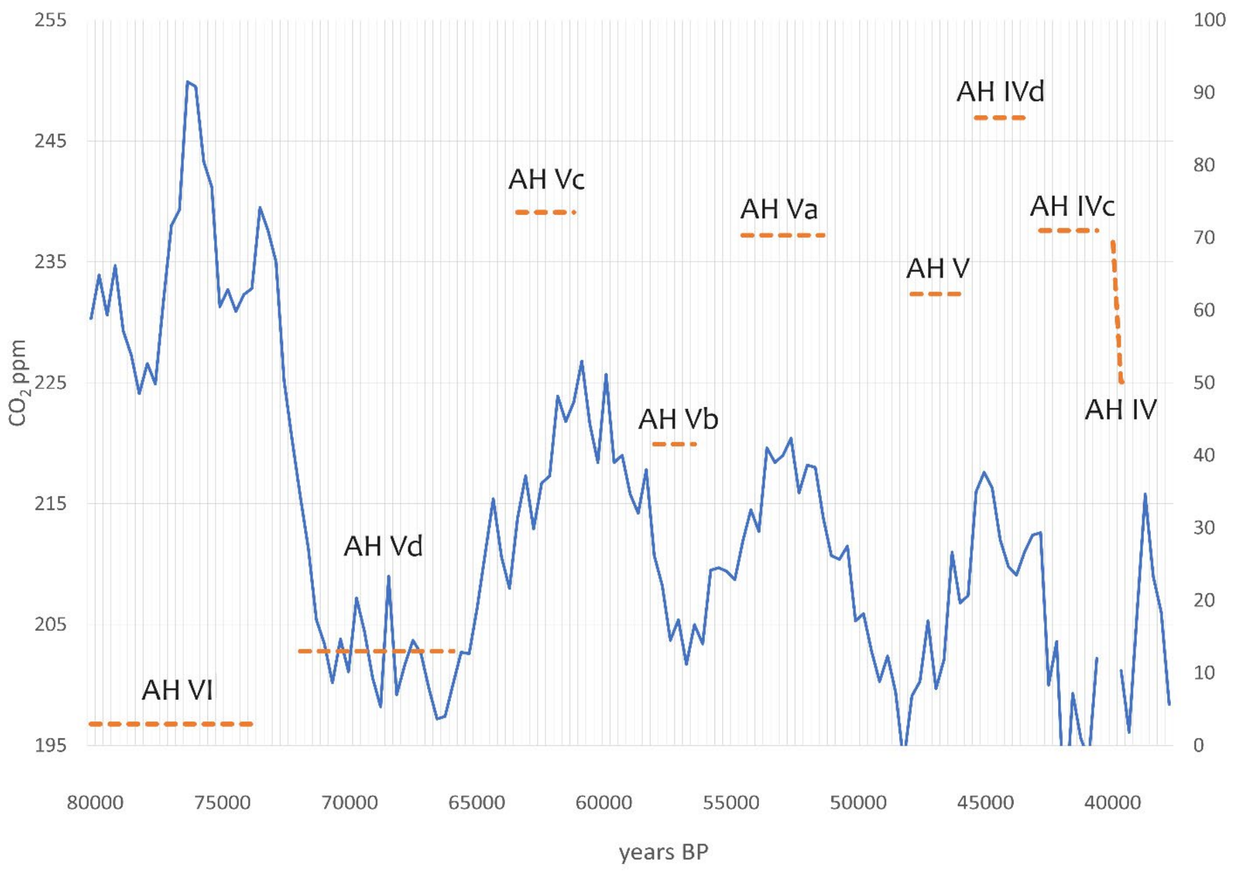


Fig. S2 Pistachio nut proportions in different archaeological horizons (AH) at Ghar-e Boof and $CO_2$ fluctuations over the last 80.000 years (raw data downloaded from NOAA.gov, data sources: 80.000-34.000 BP[2], 34.000-21.600 BP and 9.000-5.000 BP[3], 21.600 BP-9.000 BP[4]).

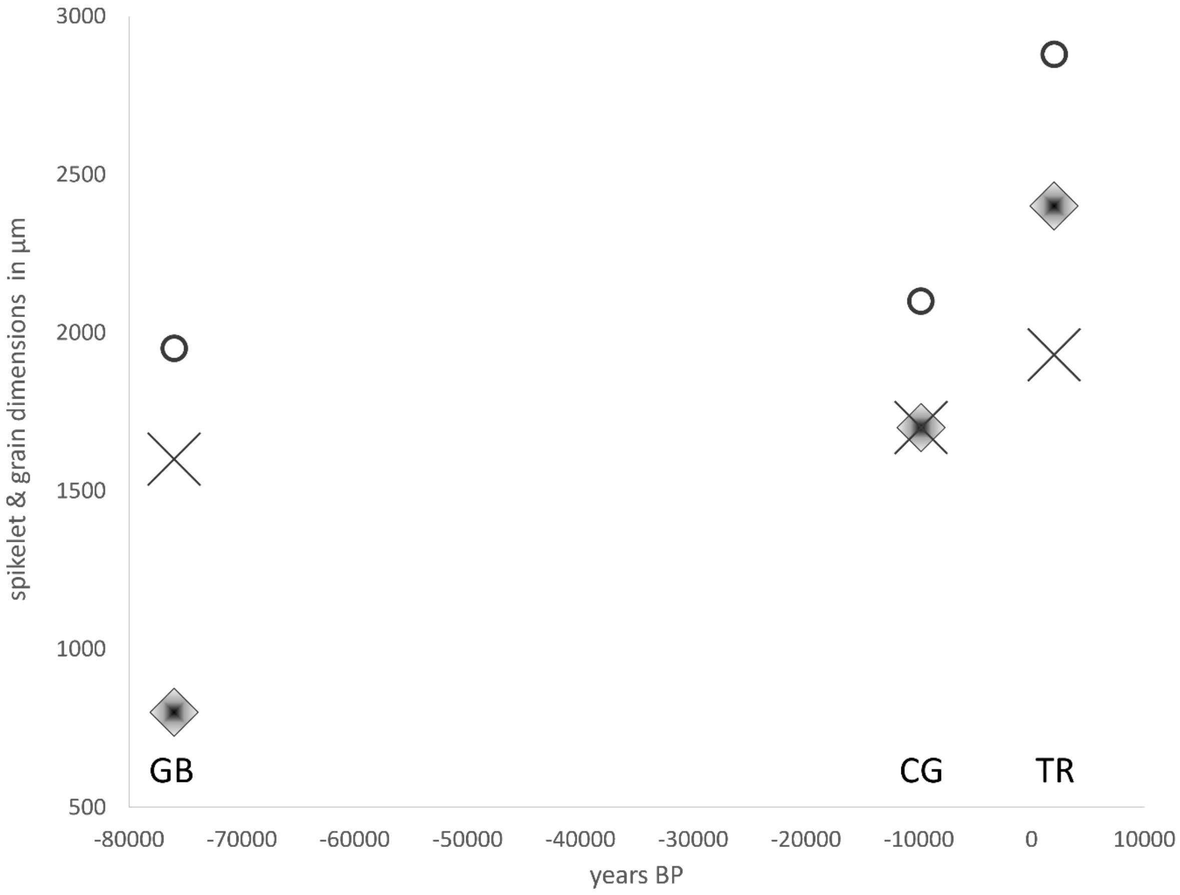


Fig. S3 Dimensions of glume wheat grains and spikelets (*Triticum boeoticum/dicoccoides*) from Middle Palaeolithic Ghar-e Boof (GB), aceramic Neolithic Chogha Golan (CG) and modern Turkey (TR; domesticated *Triticum monococcum*). Diamonds indicate spikelet width on the level of the attachment scar, circles are mean grain thickness, crosses mean grain width in µm.

# References


1 Heydari, M., Guérin, G., Zeidi, M. & Conard, N. J. Bayesian luminescence dating at Ghār-e Boof, Iran, provides a new chronology for Middle and Upper Paleolithic in the southern Zagros. *J Hum Evol* **151**, 102926 (2021). https://doi.org/10.1016/j.jhevol.2020.102926

2 Eggleston, S., Schmitt, J., Bereiter, B., Schneider, R. & Fischer, H. Evolution of the stable carbon isotope composition of atmospheric CO2 over the last glacial cycle. *Paleoceanography* **31**, 434-452 (2016). https://doi.org/https://doi.org/10.1002/2015PA002874

3 Barnola, J. M., Raynaud, D., Korotkevich, Y. S. & Lorius, C. Vostok ice core provides 160,000-year record of atmospheric $CO_2$. *Nature (London)* **329**, 408-414 (1987).

4 Monnin, E. *et al.* Atmospheric CO2 Concentrations over the Last Glacial Termination. *Science* **291**, 112-114 (2001). https://doi.org/10.1126/science.291.5501.112
